# Systematic Review of Lumbar Elastic Tape on Trunk Mobility: A Debatable Issue

**DOI:** 10.1016/j.arrct.2021.100131

**Published:** 2021-05-11

**Authors:** Robbert N. van Amstel, Karl Noten, Lara N. van den Boomen, Tom Brandon, Sven A.F. Tulner, Richard T. Jaspers, Annelies L. Pool-Goudzwaard

**Affiliations:** a Fysio Science Department, Fysio Physics Fysiotherapie, IJsselstein; b Department of Human Movement Sciences, Faculty of Behavioural and Movement Sciences, Vrije Universiteit Amsterdam, Amsterdam Movement Sciences, Amsterdam; c Department of Sports Medicine, Isala Medical Hospital, Zwolle; d Department Orthopedic Surgery, OrthoDirect Medical Clinic, Amsterdam, The Netherlands

**Keywords:** Athletic tape, Hip, Range of motion, articular, Rehabilitation, Spine, CCT, controlled clinical trial, ETA, elastic tape application, FFD, Finger Floor Distance test, FROM, flexion range of motion, LBP, low back pain, MDC, minimal detectable change, PEDro, Physiotherapy Evidence Database, RCT, randomized controlled trial, ROM, range of motion, TRM, trunk mobility

## Abstract

**Objectives:**

To systematically review the literature to analyze the effect of lumbar elastic tape application on trunk mobility, surpassing the minimal detectable change of the used outcome measurement tool, and to analyze the additional effect of applied tension and direction of elastic tape application in low back pain and participants without low back pain.

**Data Sources:**

Four databases were used: PubMed, Web of Science, Physiotherapy Evidence Database (PEDro), and Google Scholar.

**Study Selection:**

The inclusion criteria were randomized and clinical controlled trials evaluating the effectiveness of lumbar elastic tape application on trunk mobility.

**Data Extraction:**

Two researchers executed the search and a third author was consulted to resolve disagreements. The methodological quality was scored using the PEDro scale, with studies scoring ≤5 being excluded.

**Data Synthesis:**

Eight out of 6799 studies were included; 5 studied individuals with low back pain, and 3 studied participants without low back pain. Two studies scored low on the PEDro scale and were excluded. None of the reported significant changes in trunk mobility due to elastic tape application exceeded the indicated minimal detectable change. No conclusions can be drawn from the direction and applied tension of elastic tape application.

**Conclusions:**

Based on the results of this systematic review, there is no evidence supporting the effect of lumbar elastic tape application. We recommend consensus in the use of more reliable and valid instruments in future studies.

In patients with low back pain (LBP), staying active is indicated as the most important advice in rehabilitation.^1^ However, back pain reduces the ability to move freely.^2^ This adaptation is caused by altered trunk neuromuscular response in reducing the risk of noxious lumbar tissue stresses.^3^ These adaptations eventually lead to painful muscle contractions and loss in trunk mobility^4^ and are primary targets of physiotherapeutic interventions.^5^

Physical therapists regularly use elastic tape application (ETA) in treating and preventing musculoskeletal disorders.^6^^,^^7^ ETA is the application of therapeutic tape developed by Kenzo Kase in the early 1970s and has gained popularity in recent years. ETA is the gluing of an elastic cotton strip to the skin with an acrylic adhesive while the skin is stretched.

The superficial fascia within the skin is adjunct with the deep fascia and muscles owing to different types of connective tissues.^8^ The kinematic behavior of skin and fascia are unique per layer.^9^ Fascial kinematic studies showed that the skin, superficial fascia, and deep fascia will move in the cranial direction during trunk flexion and that the perimuscular fascia and muscle will move in the caudal direction during trunk flexion; the opposite occurs for trunk extension.^9^, ^10^, ^11^

Previous studies investigating possible working mechanisms and the effect of ETA have confirmed that ETA causes heterogeneous deformations of the dermis, hypodermis, and deep fascia underneath the ETA,^11^, ^12^, ^13^ increasing the blood- and lymph flow underneath the ETA and peripheral areas,^14^ enhancing proprioceptive sensation,^15^ decreasing subjective pain, increasing joint range of motion,^16^ and enhancing muscle activity.^15^^,^^17^ Moreover, it is suggested that the direction of ETA on the skin, over the muscle of interest, has specific effects on muscle activity.^18^, ^19^, ^20^ Based on these effects, using these rationales as a basis for their clinical intervention, physical therapists often use ETA to improve mobility, enhance sport and functional performance, and treat musculoskeletal complaints, including patients with LBP.^21^, ^22^, ^23^

A meta-analysis studying the effectiveness of ETA on pain and disability in a large variety of musculoskeletal complaints demonstrated moderate evidence that ETA reduces pain and very low evidence for an improvement of disability.^24^ Recently, another meta-analysis on the effectiveness of ETA in musculoskeletal disorders on pain and disability, which only included studies comparing ETA with a sham condition, showed that pain does not immediately change posttreatment. However, pain does reduce during follow-up (range, 4-12wk).^25^

Although it seems logical in patients with LBP to study the effect of ETA on pain and disability, we expected the largest effect to occur in the gain in trunk mobility (TRM) based on the above-described working mechanisms. Some studies have published the TRM outcome; however, this outcome was not included in the meta-analyses studies. Furthermore, in the studies published demonstrating a positive effect, the minimal detectable change (MDC) of the measurement tool used to measure mobility was not considered. The purpose of this study was to systematically review the literature to analyze the effect of ETA on TRM surpassing the MDC of the outcome measurement tool, and if effective, to analyze the additional effect of applied tension and direction of ETA in patients with LBP with respect to participants without LBP.

## Methods

### Protocol and registration

This systematic review adhered to the Preferred Reporting Items for Systematic Reviews and Meta-Analyzes guidelines^26^ and was prospectively registered in the Open Science Framework (https://osf.io/2jhzg). The analyses were based on cumulative data from previously published studies, so no ethical approval was required.

### Search strategy, inclusion, and exclusion of studies

A systematic literature research was performed independently by 2 reviewers (R.A. and L.B.). In the case of disagreement, a third independent reviewer was consulted. Publications were identified by searching multiple literature databases, including PUBMED, Web of Science, Physiotherapy Evidence Database (PEDro), and Google Scholar. To exclude gray literature, we decided that only the first 400 Google Scholar results would be screened.^27^ A sensitive search string was used (table 1). The search was performed between September and December 2019 on “all fields”; the filter “humans” was applied if possible.

**Table 1:** Databases and algorithms for the literature search

Database	Search Algorithm
PubMed (n=712)	(Range of motion[MeSH Terms]) AND Low back[MeSH Terms]) OR Spine[MeSH Terms]) OR Lumbar) OR Trunk) AND Elastic tape) OR Elastic taping) OR Kinesio tape) OR Kinesio taping) OR Kinesiotaping AND Humans
Web of Science (n=541)	Range of motion (TS) AND Low back (TI) AND Elastic tape OR Elastic taping (TI) OR Kinesio tape (TI) OR Kinesio taping (TI) OR Kinesiotaping (TI)
Scholar (n= 5540)	(Range of motion) AND (Low back pain) AND (Elastic taping OR Kinesio Tape OR Kinesio taping OR Elastic tape) AND (Human)
PEDro (n=6)	Range of motion AND Low back OR Spine OR Lumbar AND Elastic tape OR Elastic taping OR Kinesio tape OR Kinesio taping OR Kinesiotaping

NOTE. Diverse combinations were made with the following keywords: Range of motion, Low back, Spine, Lumbar, Trunk, Elastic tape, Elastic taping, Kinesio tape, Kinesio taping, and Kinesiotaping.

#### Study selection

In advance, narrative research was performed by 2 authors (R.A., K.N.) into the psychometric quality of various mobility measurement tools (table 2) to only include studies using reliable and valid instruments to measure TRM in terms of trunk range of motion (ROM) and to register the minimal detectable difference per outcome measure. Based on the quality of the instruments, studies were excluded using a mobile inclinometer application not reaching sufficient psychometric quality. Smartphones have a great range in sensor and software quality, which influence the reliability and validity of the smartphone range of motion applications.^39^

**Table 2:** Error parameter

Measure Tool	Direction	ICC^a^	brxy	SEM	MDC	Studies
FFD test	Flexion	0.99	*	*	MDC_95_= 9.80^a^	Robinson and Mengshoel^28^ and Ekedahl et al^29^
Schober test	Flexion	0.95	*	*	MDC_95_=1.80^a^	Robinson and Mengshoel^28^ and Tousignant et al^30^
	Extension	0.76	*	0.93^a^	MDC_95_=2.60^ac^	Williams et al^31^
Inclinometer	Flexion	0.96	*	*	MDC_90_=7^a^	Kolber et al^32^
	Extension	0.88	*	*	MDC_90_=6^a^	
	Lateral flexion (right)	0.96	0.96	1.17^a^	MDC_95_=3.2^ac^	Ng et al^33^
	Lateral flexion (left)	0.92	0.94	1.68^a^	MDC_95_=4.7^ac^	
	Rotation (right)	0.96	0.96	1.79^a^	MDC_95_=5.0^ac^	
	Rotation (left)	0.95	0.94	1.99^a^	MDC_95_=5.5^ac^	
BBROM device flexion	Flexion	0.94-0.95	*	*	MDC_95_=2.16-2.83	Phattharasupharerk et al^34^ and Kachingwe et al^35^
	Extension	0.98-0.99	*	*	MDC_95_=1.64-2.30	
Electrogoniometer	Flexion	0.98	0.96-0.99	0.35-0.38^b^	MDC_95_=0.97-1.05^bc^	Paquet et al^36^
Seat and reach test	Flexion	0.97	r=0.89-0.98	*	MDC_95_=4.0^a^	López-Miñarro et al^37^ and Hui et al^38^
Back-saver-sit-and-reach	Flexion (right leg)	0.97	r=0.89-0.98	*	MDC_95_=3.0^a^	
	Flexion (left leg)	0.96	r=0.89-0.98	*	MDC_95_=4.0^a^	

NOTE. ^ac^SEM=^SD^√1-ICC is based on reliability, ^bc^SEM=^SD^√1-rxy is based on validity, MDC_95_= SEM × 1.96 × √2, is error-parameter based on ^ac^ or ^bc^. If it was necessary, the MDC_95_ was calculated using ICC or rxy.

Abbreviations: ICC, intraclass correlation coefficient; rxy, Pearson.

⁎ No information known.

Inclusion criteria were randomized controlled trials (RCTs) and controlled clinical trials (CCT) on the effects of ETA on TRM in participants without LBP and patients with LBP. Only trials with a control group were considered eligible (eg, ETA vs placebo/sham ETA and ETA vs no-taping intervention or usual care). ETA had to be applied to the individual's back with no restriction to the manor, technique, direction, or applied tension. Studies were included if TRM was a (primary) outcome measure with methods used according to table 2. No restrictions were applied to the search strategy regarding the date of publication. Studies written in Dutch, English, or German were included.

#### Data extraction

The first (R.A.) and third (K.N.) authors performed a systematic literature search independent from each other. The titles, abstracts, and full texts (indicated) of all articles were screened for inclusion by the reviewers. Data were extracted independently by the first (R.A.) and second (L.B.) reviewers from full-text articles. Data extraction included details on the ETA protocol used (eg, taping method, tape application direction, amount of stretch applied to the tape) and the effect on the TRM.

#### Standard measurement error

All calculations for measurement error parameters were performed with MATLAB.^a^ Per instrument, the MDC was retrieved as an error parameter to analyze the meaningfulness of the significant improvements found in the systematically included study-results. If the MDC error parameter was absent, the SEM_consistency_ was calculated with use of the interobserver reliability $\left({\sqrt[{SD}]{1-ICC}}^{SD}\right)$ or the Pearson correlation validity $\left(\sqrt[{SD}]{1-r}\right)$. Subsequently, it was used to calculate the MDC to determine the magnitude of change that would exceed the threshold of measurement error at 95% confidence interval $\left(1.96\times SEM\times \sqrt{2}\right)$ (see table 2).

#### Quality assessment

The methodological quality of the included RCTs and CCTs was assessed using the 11-item PEDro scale. The RCTs and CCTs had to compare at least 2 interventions, and one intervention had to lay within the scope of this systematic review. The PEDro scale has been described as a valid and reliable tool for the investigation of the internal validity of RCTs and has shown sufficient reliability for use in systematic reviews.^40^, ^41^, ^42^ Full texts that met the eligibility criteria were independently assessed using the PEDro scale for methodological quality by the first and second reviewer (R.A., L.B.). In case of disagreement, a third independent reviewer was consulted for the final score. Studies scoring ≤5 on the PEDro scale were excluded owing to their low methodological quality that indicates they are less likely to yield meaningful results.^43^

## Results

### Description of studies

**Fig 1:**
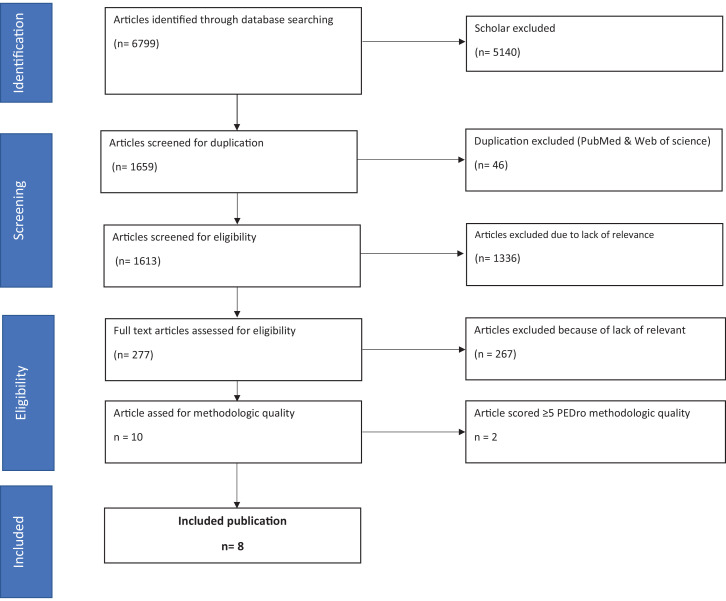
Preferred Reporting Items for Systematic Reviews and Meta-Analyses flowchart summarizing the yield of the search strategy and screen procedure.

In total, 6799 titles were found in the initial search (fig 1). Only the first 400 Google Scholar results were included (5140 were excluded).^27^ Upon eliminating duplicates, the remaining 1613 titles and abstracts were reviewed, excluding another 1336 studies and determining eligibility for the remaining 277 articles. Of these 277 articles, 267 articles were omitted owing to a lack of relevant information (eg, no ROM outcome, no ETA research, and/or ETA in combination with another intervention), resulting in 10 studies that met the requirements for methodological evaluation.^44^, ^45^, ^46^, ^47^, ^48^, ^49^, ^50^, ^51^, ^52^, ^53^ After the methodological evaluation, 2 studies did not meet our quality criteria of a score of ≤5 on the PEDro scale and had to be excluded,^52^^,^^53^ which resulted in 8 articles that met the criteria for inclusion.

There was a good literature search agreement between the authors who reviewed the literature (ĸ=0.736; *P*>.001).^54^^,^^55^ The 8 studies included were published between 2007-2019 and included a total of 369 individuals. Patients with LBP (n=236) were analyzed in 6 studies, and individuals without LBP (n=133) were analyzed in 3 studies. The per-study sample sizes ranged from 20-75 individuals.

### Methodological quality

Nine of the included studies analyzed the ETA effect on the TRM by RCT, 2 studies analyzed this by CCT. The external validity was guaranteed in 7 studies^44^, ^45^, ^46^, ^47^, ^48^^,^^50^^,^^51^ while this was not guaranteed in 3 studies.^49^^,^^52^^,^^53^ The internal validity was scored “excellent” in 6 studies.^44^, ^45^, ^46^^,^^48^^,^^50^ Moreover, 2 studies were scored as “good,”^47^^,^^49^^,^^51^ 1 study was scored as “fair,”^53^ and 1 study was scored as “poor.”^52^ We excluded the last 2 studies from this review owing to their low quality because they might provide unmeaningful information concerning ETA effects on the TRM (table 3).

**Table 3:** Summarized PEDro scores

Study	External Validity	Internal Validity:Present Criteria on PEDro Scale										Score	Quality	Strength, % (score)
	1	2	3	4	5	6	7	8	9	10	11			
Al-Shareef et al^44^	Yes	+	+	+	+	-	+	+	+	+	+	9/10	Excellent	81 (9/11)
Castro-Sánchez et al^45^	Yes	+	+	+	+	-	+	+	+	+	+	9/10	Excellent	81 (9/11)
Grzeskowiak et al^46^	Yes	+	+	+	+	-	-	+	+	+	+	8/10	Excellent	72 (8/11)
Lemos et al^47^	Yes	+	-	-	+	-	-	+	+	+	+	6/10	Good	54 (6/11)
Preece and White^48^	Yes	+	+	+	+	-	-	+	+	+	+	8/10	Good	72 (8/11)
Shin and Heo^49^	No	+	-	+	+	-	-	+	+	+	+	7/10	Good	63 (7/11)
Velasco-Roldán et al^50^	Yes	+	+	+	+	-	+	+	+	+	+	9/10	Excellent	81 (9/11)
Norman et al^51^	Yes	+	-	+	+	-	-	+	+	+	+	7/10	Good	63 (7/11)
Yoshida and Kahanov^52^	No	-	-	-	-	-	-	-	+	+	-	2/10	Poor	18 (2/11)
Nawrot et al^53^	No	-	-	+	-	-	-	+	+	+	+	5/10	Fair	45 (5/11)

NOTE. 9-10 points indicates excellent quality, 6-8 points indicates good quality, 4-5 points indicates fair quality, and 0-3 points indicates poor quality.^40^ Each criterion equals 1 point for a possible total of 10 points. The criterion content are: (1) clear inclusion and clear exclusion criteria; (2) random allocation/concealed allocation; (3) baseline comparability; (4) blinded assessors; (5) blinded participants; (6) blinded therapist; (7) adequate follow-up; (8) variable measured in >85% of participants; (9) between group comparisons; and (10) points estimates and variability.

### Differences in study design

All studies used the ETA in the experimental group and compared it with a control group (sham ETA intervention or nonintervention). However, there was a difference in study design. Some studies studied the effectiveness of ETA on the TRM in situ (condition 1), but others evaluated the effect after ETA was removed (condition 2). Two of the 8 studies evaluated condition 1,^48^^,^^49^ 3 of the 8 studies evaluated condition 2,^44^^,^^51^^,^^56^ and 3 studies evaluated the effect of both conditions.^45^^,^^47^^,^^50^ The characteristics of the 8 studies included in the systematic review are shown in table 4.

**Table 4:** Results and conclusions of studies of elastic tape application on the trunk ROM

Study and Conditions	No. of Participants	Intervention	Intervention Duration	Assessment Method	Results: Condition 1 (ETA in situ)	Results: Condition 2 (ETA Treatment Effect)	MDC+/-		Conclusion
Preece and White^48^ RCT, nonspecific chronic LBP	34 without LBP	Experimental set-up: The paravertebral muscles I-shaped ETA technique was used in the EGr. Two I-strips were used (5 cm wide and ~25 cm long) and were applied bilaterally with 10%-15% tension on the individual's back. The ETA was applied from the PSIS to level T8. The anchor was placed at the height of the PSIS in a standing position and adhered in comfortable flexion.	• One session ETA • ETA immediately posttested	MFFD test • Flexion ROM •Tested with ETA in situ	Description (mean±SD): • EGr: Baseline: 26.83±10.95 After: 24.08±11.05 • CGr: Baseline: 28.18±11.39 After: 26.60±9.94				Directly after ETA was tested in situ, there was a significant effect found in the EGr on the flexion ROM with ETA in situ. However, the results are not reliable regarding the MDC. Hence, ETA in CON1 does not influence flexion trunk mobility and is not better than paravertebral-sham tape.
		Control set-up: The CGr received sham “I” horizontal L4 elastic tape with 0% tension.			Within time (mean difference±SD): EGr: Baseline-after MD=2.75±2.59 95%CI: 1.54-3.96 *P*<.05 –CGr: Baseline-after MD: 1.57±2.87 95%CI: –0.09 to 3.23 *P*=NS		- *		
					Between groups (baseline chanced difference) • Baseline-after groups d: MD: 1.57±2.87 95% CI: –0.09 to 0.75 *P*=NS • EGr=CGr (Correction for age): *P*=NS EGr=CGr				
Shin and Heo^49^ Participants without LBP	60 participants without LBP	Experimental set-up: In this study, the EGr received 2 types of ETA techniques at the same time. *Tape 1:* ligament ETA: 1 I-shaped ETA (5 cm wide and ~20 cm long) was used. The tape was applied on the participant's back from the PSIS to the opposite PSIS in standing position. After the application of the SI joint ET, the second tape technique (tape 2: paravertebral) was applied.	One session ETA • ETA attached in situ for 30 minutes	BROM II: • Flexion ROM • Extension ROM • Tested with ETA in situ • Inclinometer: • Lateral-FROM • Rotation ROM • Tested with ETA in situ	Description: (mean±SD): EGr: Baseline flexion: 32.57±4.08 After flexion: 26.83±4.62 Baseline extension: 11.43±3.26 After extension: 11.20±3.29 Baseline r-latroflexion: 32.33±3.93 After r-latroflexion: 27.47±4.45 Baseline l-latroflexion: 33.10±3.63 After l-latroflexion: 28.17±3.39 Baseline r-rotation: 35.30± 5.90 After r-rotation: 29.80±6.33 Baseline l-rotation: 37.03±4.04 After l-rotation: 31.57±5.71				One session of ETA for 30 minutes and measured in situ does significantly affect the flexion, lateral-flexion, and rotation ROM. Regarding the MDC, the flexion, lateral-flexion, and right rotation ROM evolutions were meaningful. Hence, ETA in CON1 does influence flexion, lateral-flexion, and right rotation trunk mobility and is better than X-sham elastic tape application. However, the left rotation and extension trunk mobility did not improve and is not better than X-sham tape.
		*Tape 2: paravertebral* muscles ETA: 2 I-stripes were used (5 cm wide and ~30 cm long) and were applied paravertebral at the height of the erector spinae started from ~L5/S1 to the area below the scapula.			Within time (mean difference±SD):				
		Control set-up: The GRr received sham tape, which was applied in an X-shape that crossed around T12.			EGr: Flexion ROM : MD=5.74±SEP1.33 *P*<.05 EGr: EROM Baseline-after=NS EGr: R-LFROM: Baseline-after MD=4.86±SEP1.38 *P*<.05 EGr: L-LFROM Baseline-after MD=4.93±SEP1.05 *P*<.05 EGr: R-RROM: Baseline-after MD= 5.50±SEP2.41 *P*<.05 EGr: L-RROM: Baseline-after MD= 5.46±SEP1.82 *P*<.05 CGr: Baseline- Post difference *P*=NS		+ * + + + - *		
					Between groups: EGr-CGr: Baseline-post changed value *P*<.5				
Al-shareef et al^44^ RCT, nonspecific chronic LBP	44 LBP	Experimental set-up: The paravertebral muscles I-shaped ETA technique was used in the EGr. Two I- stripes were used (5 cm wide and ~20 cm long) and were applied bilaterally with 10%-15% tension on the patient's back. The initial anchor point of tape (4-5 cm) was applied to the posterior superior iliac crest without tension up to T12. The tape was applied in the flexion position, and the participants were asked to bend forward during the application where the tape has adhered to the muscles along.	• Twice a week treatment • Total of 4 sessions • ETA attached in situ until next intervention • Follow up at 4 weeks	Modified Schober's test: • Flexion ROM • Tested without ETA in situ (after 4 weeks)		Description: EGr: Baseline: 4.42±0.40; W2: 6.27±0.41; W4 6.27±0.41 CGr: Baseline: 4.20±0.61; W2: 5.31±0.68; W4: 5.31±0.68			Twice a week treatment of a total of 4 sessions significantly influences the flexion ROM. However, based on the MDC, the evolution is not meaningful after 2 sessions, but it is after 4 sessions. Hence, ETA in CON1 does not influence trunk flexion trunk mobility after 2 ETA sessions and is not better than paravertebral-sham elastic tape application. However, it does improve the flexion trunk mobility after 4 ETA sessions.
		Control set-up: CGr received 2 shams “I” paravertebral elastic tape with 0% tension.				Within time: EGr: Baseline-W2: MD=–1.32±0.24; *P*<.05 EGr: Baseline-W4: MD=1.85±0.23; *P*<.05 CGr: Baseline-W2: MD=–0.61±0.20; *P*<.05 CGr: Baseline-W4: MD=–1.11±0.23; *P*<.05	- + - -		
						Between groups: (Pair difference): Baseline-W2: Groups d: MD=–0.71; 95% CI=–-0.85 to –0.56; *P*<.05 EGr>CGr	-		
						Baseline-W4: Groups d: MD=–0.73; 95% CI=–0.88 to –0.58; *P*<.05 EGr > CGr	-		
Norman et al^51^ RCT, nonspecific chronic LBP	20 LBP	Experimental set-up: The paravertebral muscle technique. Two I-strips (5 cm wide and ~20 cm long). The ET was applied with ~15% tension on the participant's back. ETA was adherend to the skin above the paravertebral muscles from sacrum to the level T12. The tape was applied in a flexion position, and the participants were asked to bend forward during the application where the tape adhered along to the muscles.	• One session ETA • ETA attached 7 days in situ	Schober's test: • Flexion ROM • Tested without ETA in situ (after 4 weeks)		Description: EGr: Baseline: 20.7±0.46 W2: 21.0±0.37 W4: 20.6±0.59 CGr: Baseline: 20.8±.57 W2: 20.6±0.60 W4: 20.6±0.63			One session of ETA for 7 days in situ has a significant treatment effect on the flexion ROM, only in the first 2 weeks and not after 4 weeks. Based on the MDC parameters, the evolution after week 2 is not meaningful. Hence, ETA in CON2 does not have a significant treatment effect in terms of improved flexion trunk mobility and is not better than no treatment.
		Control set-up: The CGr did not receive any intervention.				Within time: EGr: Baseline-W2: MD=.30±SEP.16; *P*<.05 EGr: Baseline-W4; *P*=NS CGr: Baseline-W1; *P*=NS CGr: Baseline-W4; *P*=NS	* * *		
						Between groups: (Pair difference): EGR-CGR: Baseline-W1; *P*<.05 EGR-CGR: Baseline-W4; *P*=NS	*		
Grzeskowiak et al^46^ RCT, lumbar disc herniation (L4 and/or L5)	38 LBP	Experimental set-up: A thoracolumbar fascia ETA technique was used. Two I-stripes were used (5 cm wide and ~30 cm long), each extending from the posterior axillary fold of 1 side to the greater trochanter of the opposite side of the body, were applied to form X- shaped application with midpoint overlapped over the lumbosacral junction. The midpoint was attached with no tension, and the tails of stripes were applied directly to the skin with paper-off tension (~15%–25% of tapes stringing). The tails were oriented in the direction of fibers of the superficial lamina of the posterior layer of TLF (~40 degrees craniolateralto-caudomedial).	• One session ETA • ETA attached in situ for 7 days	Electrogoniometer: • Flexion ROM • Tested without ETA in situ (after 7 days)		Description: EGr: Baseline: 51.8±10.7 After 7 days: 52.9±9.1 CGr: Baseline: 53.6±11.4 After 7 days: 54.3 ±12.7			After 7 days of ETA in situ, there was no significant treatment effect found on the flexion ROM after the ETA was removed. In conclusion, ETA in CON2 has no treatment effect in terms of improved flexion and extension trunk mobility and is not better than rigid paravertebral tape application.
		Control set-up: The CGr received 2 “I” shaped rigid tape strips, which were applied using the same protocol as in the CGr.				Within time: EGr: Baseline-after: MD, *P*=NS CGr: Baseline-after: MD, *P*=NS	*		
						Between groups Not significant	*		
Castro-Sánchez et al^45^ RCT, nonspecific chronic LBP	59 LBP	Experimental set-up: In the study, the star elastic tape application technique was used. The Star application comprises multiple use ligament ETA. Four I-strips were used (5 cm wide and ~25 cm long) and were adherend on the participant's back with 25% tension. The 4 I-strips overlap in a star shape over the point of maximum lumbar pain. The anchors of the tape were applied without tension.	• One session ETA • ETA attached in situ for 7 days	Inclinometer (Fleximeter): • Flexion ROM • Extension ROM • Tested with ETA in situ (in week 1) • Tested without ETA in situ (after 5 weeks)	Description (mean±SD):	Description:(mean±SD):			One session of ETA for 7 days in CON1 and CON2 does significantly affect the flexion ROM. Based on the MDC, evolution after weeks 1 and 5 are not meaningful. ETA in CON1 and CON2 does not influence trunk flexion - and extension trunk mobility and is not better than sham elastic tape application.
					Flexion ROM inclinometer EGr: Baseline: 94±7 W1: 98±7 CGr: Baseline: 90±9 W1: 92±11	FROM inclinometer EGr: W5: 97±7 CGr: W5: 94±8			
		Control set-up: The CGr received 1 horizontal I- Sham ETA (25% tension).			Within time (mean difference±SD):	Within time: (mean difference±SD):			
					EGr: Baseline-W1: MD= 5±2; *P*<.05	EGr: Baseline-W5: MD=4±2; *P*<.05	-	-	
					CGr: Baseline-W1: MD=2±7; *P*=NS	CGr: Baseline-W5: MD=4±8; *P*<.05	-	-	
					Between groups (Pair difference):	Between groups (Pair difference): EGr-CGr: Baseline-W5: Groups d: MD=–0.1, 95% CI=–3.1 to 2.8; *P*=NS EGR=CGR			
					EGr-CGr: Baseline-W1: MD=2.6, 95% CI=0.0-5.2; *P*>.05				
					EGr>CGr		*		
Lemos et al^47^ Female participants without LBP (BMI >29)	39 without LBP	Experimental set-up: The paravertebral muscles I-shaped ETA technique was used as an experiment. Two types of ETA techniques were tested. Both ETA techniques used 2 I-strips (5 cm wide and ~30 cm long) which were applied on the participant's back during maximal trunk flexion.	• One session ETA • ETA attached in situ for 48 hours	FFD test: • Flexion ROM • Tested with ETA in situ (48 hours later) • Tested without ETA in situ (after 30 days) Schober's test: • Flexion ROM • Tested with ETA in situ (24 hours later) • Tested with ETA in situ (48 hours later) • Tested without ETA in situ (after 30 days)	Description (mean±SD):	Description:(mean±SD)			There is conflicting evidence between 2 different
					FFD test EGr1: Baseline: 22.37±2.63 After 48 hours: 13.88±1.66 EGr2: Baseline: 17.05±1.89 After 48 hours: 11.74±2.14 CGr: Baseline: 16.73±2.63 After 48 hours: 16.54±2.78	FFD test EGr1 Follow-up 30 days: 15.53±1.99 EGr2 follow-up 30 days: 13.51±1.99 CGr: Follow-up 30 days: 15.85±2.79			Flexion ROM measure instruments on the ETA in CON 1 and CON2. However, the FFD did detect a significant ETA effect in CON1 as in CON2. However, concerning the MDC, the evolution after 48 hours and at follow-up at 30 days are not meaningful.
									Hence, ETA in CON1 and CON2 does not significantly affect the flexion trunk mobility and is not better than no treatment.
		ETA 0% tension: In EGr1, the ETA was applied with 0% tension.							
		Fascia correction technique: The ETA in EGr2 was applied using short and long oscillation load to the tape to add a varied amount of tension between 15% and 50%.			Within: EGr1: Baseline-after 48 hours: *P*<.05 EGr2: Baseline-after 48 hours: *P*<.05	Within time: EGr1: Baseline-after 30 days: MD=6.84±SEP1.03; *P*<.05 EGr2: Baseline-after 30 days: MD=3.54 ±SEP.83; *P*<.05	-	-	
							-	-	
		Control set-up: The CGr did not receive any intervention.			Between groups: • Not significant	Between (ETA in situ and ETA effect): EGr1: After 45u–after 30 days: *P*=NS EGr2: After 45u-after 30 days: *P*=NS	*		
					Others: Schrober's test tape in situ: *P*=NS	Others: No significant changes found for: CGr Schober's test: after 25 hours and 30 days Between EGr1, EGr2, and CGr	*		
Velasco-Roldán et al^50^ RCT, nonspecific chronic LBP	75 LBP	Experimental set-up: In this study, 3 types of ETA methods were analyzed. All ETA interventions used the paravertebral I muscle technique. Two I-stripes (5 cm wide and 30 cm long) were applied on the participant's back. The ET was applied as follows: the initial anchor point of tape was applied at the height of the sacrospinalis without tape tension. The participant was asked to perform gradual trunk flexion, while the rest of the tape was adhered on the paravertebral muscles.	• One session, ETA • ETA attached in situ for 24 hours	FFD, Sit and reach test, Back saver sit and reach: • Flexion ROM • Tested with ETA in situ (10 minutes later) • Tested with ETA in situ (24 hours later) • Tested without ETA in situ (after ~24 hours)	Description:	Description:			
					Left Back saver sit and reach test EGr 2: Baseline: 26.63±8.9 After 24 hours: 28.83±7.9 EGr 3: Baseline: 27.75±7.5 After 24 hours: 29.11±6.9	Left Back saver sit and reach test EGr1: After 24 hours ETA removed: 30.03±7.6 EGr2: After 24 hours ETA removed: 30.31±7.1 EGr3: After 24 hours ETA removed: 30.21±7.7			
									ETA in situ and after it was removed does not significantly affect the flexion ROM after it was worn for 24 hours. There was a significant difference found for the different ETA tensions. However, concerning the MDC, the difference between the ETA tension was not meaningful. Hence, ETA in CON1 and CON2 with different ETA tensions does not improve flexion trunk mobility Moreover, there is no superior ETA to prescribe.
					Within time: NS	Within time: Baseline-after 25 hours: *P*=NS			
		EGr1: The tape was applied with 15%-20% tension until the T12 level was reached. This was repeated for the other side.							
		EGr2: The tape was applied with 40% extra tension until the T12 level was reached. This was repeated for the other side.							
		Control set-up EGr3: The tape was reduced to 0% before it was adhered to the skin over the muscles.			Between group pre-post difference: EGr2-EGR3: Baseline-after 24 hours: MD=0.84; *P*<.03	Between: NS	-	* *	
					Others: No significant changes found for ETA in situ: Finger-to-floor test Sit and reach test	Others: No significant changes found for ETA effect: Finger-to-floor test Sit and reach test	*		

Abbreviations: BMI, body mass index; CGr, control group; CI, confidence interval; CON1, ETA tested with ETA in situ; CON2, ETA removed before the tests are run; EGr, experimental group; MD, mean difference; MFFD, Modified Finger Floor Distance; NS, not significant; PSIS posterior-superior iliac spine; SEP, pooled Satterhwaite of deviation; SI, sacroiliac; W2, week 2; W4, week 4.

⁎ Not significant

### Flexion ROM

There is conflicting evidence with regard to the effectiveness of ETA on TRM. Regarding condition 1 with ETA in situ, none of the control groups showed a significant change in flexion ROM (FROM). Two included studies did not find a significant improvement in FROM in patients with LBP.^48^^,^^50^ In contrast, 3 studies found a significant improvement in FROM in patients with LBP ^45^^,^^47^^,^^48^ and 2 studies found a significant improvement in FROM in individuals without LBP^47^^,^^49^ regarding condition 1 ETA. Two of these studies concluded that ETA affected the FROM positively measured with the Finger Floor Distance test (FFD).^47^^,^^48^ Preece and White^48^ concluded that this FFD improved immediately in patients with LBP after the ETA was applied. In addition, Lemos et al^47^ concluded the same in individuals without LBP after the ETA was worn for 48 hours. Both studies did not exceed the threshold of FFD measurement error at a 95% confidence interval using the MDC_95_ of 9.8 cm.^28^ Based on the MDC_95_, the significant change reported in these studies did not surpass the MDC.^47^^,^^48^ One study reported a positive effect of ETA on the FROM with an inclinometer (fleximeter) after the ETA was worn for 1 week. In this study, a significant evolution was found between baseline and the 1-week follow-up.^45^ The inclinometer MDC_90_ indicated that the observed change in this study was made within the intraobserver error (MD=5±SD2<MDC_90_=7). The significant change in this study cannot be considered as a true change.^32^ Shin and Heo^49^ found a significant FROM evolution in individuals without LBP measured with a BROM II device, reporting that it was beyond its MDC_95_ (MD=5.74±SD1.33>MDC_95_=2.83).^34^

Also, conflicting evidence is present in condition 2. Regarding the evaluated effectiveness of ETA on FROM in condition 2, 3 studies failed to demonstrate significant FROM change in patients with LBP,^50^^,^^51^^,^^56^ and 1 study in individuals without LBP after ETA was removed.^47^ By contrast, 3 studies reported a significant change in condition 2 for patients with LBP^44^^,^^45^^,^^51^ and 1 in individuals without LBP.^47^ Two of these studies evaluated the effectiveness of ETA using the Schober flexion test. In both studies, the FROM increased positively from baseline to week 2.^44^^,^^51^ The demonstrated significant difference in both studies did not exceed the MDC_95_ measurement error of 1.8 cm.^28^ The third study reported a significant effect in patients with LBP on FROM measured with an inclinometer,^45^ although the change was not beyond the MDC_90_ (MD=4±SD2<MDC_90_=7).^33^ Finally, the last study reporting a significant FROM change measured with the FFD after 2 types of ETA treatment sessions of 48 hours^47^ also did not surpass the MDC (MD=6.84±SD1.03<MDC_95_=9.8>MD=3.54±SD.83).^28^

### Extension ROM

No study found a significant difference regarding the extension ROM.

### Lateral flexion and rotation ROM

One study measured the lateral-flexion and rotation ROM in individuals without LBP. Both were evaluated with an inclinometer. Both lateral-flexion ROM and rotation ROM directions significantly changed in condition 1 when the ETA was worn for 30 minutes. However, the baseline-to-post difference in the left rotation ROM was not beyond the MDC_95_ and cannot be taken as a meaningful evolution (MD=5.46±SD1.82>MDC_95_=5.5).

### Tape direction and applied tension

Six out of 8 studies evaluated the caudal to cranial paraspinal placement of the ETA effect and no other placement.^44^^,^^47^, ^48^, ^49^, ^50^, ^51^ The ETA tension varied between approximately 0%-50%. The effects of differences in tensions on TRM effect was evaluated in 4 studies.^44^^,^^47^^,^^48^^,^^50^ The ETA tension difference did not exceed the MDC_95_ and was deemed irrelevant.

## Discussion

This systematic review demonstrates that ETA does not affect TRM in individuals with and without LBP. Those studies that reported a significant positive effect of ETA on TRM did not consider the MDC of the outcome instrument.^44^^,^^45^, ^46^, ^47^, ^48^^,^^51^ None of the studies included in this systematic review surpassed the MDC in their results, and none of the measurement tools were precise and accurate.

The quality of the included RCTs, assessed by PEDro score, was generally high, despite the small sample size in all studies. However, the methodological quality scored by PEDro is based on the design and does not account for the psychometric quality of the mobility measurement instruments used. Hence, in this systematic review, we used the MDC to correct for this.

No conclusion can be drawn regarding the effects of ETA direction because none of the included studies investigated the effect of the ETA direction on TRM. This is surprising because the opposite direction of ETA application has been described to have an effect.^17^, ^18^, ^19^, ^20^^,^^57^ However, most (6 of 8) of the studies applied the ETA from caudal to cranial over the lumbar paravertebral muscles and did not compare the effect of this direction with that of the opposite (cranial to caudal) ETA direction. Moreover, the resistance in elastic tape is determined by the Youngs's modulus of the tape and tension that is applied to the tape.^58^ The studies that were included in this review used variable types of tape brands and, subsequently, the applied tension differed between studies. According to a biomechanical engineering study, each brand of elastic tape creates a different level of tension at equivalent strain conditions and as such, the ETA tension could be interindividual depending on body characteristics and skin flexibility.^59^ Hence, the variability in tension may be an explanation for the lack of a meaningful effect.

In general, the elastic tape applications used in the studies are not similar to those applied in clinical practice since longitudinal paravertebral, diagonal thoracolumbar fascia, and horizontal elastic tape applications are used.^60^, ^61^, ^62^ Moreover, different ETA directions are tested before ETA in choosing the best ETA direction.^63^

### Study limitations

The strength of this study is that narrative research was done in advance into the psychometric quality of various mobility measurement instruments to analyze the effectiveness of ETA. The validity, reliability, and MDC were retrieved from these studies. If the MDC was absent, the SEM and MDC_95_ were calculated. Subsequently, the strictest MDC error-parameter was used to evaluate the meaningfulness of the reported significant changes in TRM influenced by ETA. We excluded studies in which a mobile phone inclinometer application was used to evaluate the effect because of the insufficient reliability and the various downloadable applications. Among RCTs and CCTs, only trials with a control group were considered eligible. However, we excluded RCTs and CCTs that used ETA in a multimodal interventional setting because of the possibility that the ETA could interact with the other interventions used.

There are also limitations in this systematic review. In general, the included studies are not homogeneous concerning the study population. Moreover, different evaluation time points were used to evaluate the TRM effect and there are unstandardized or unknown standard measure procedures, failed sham elastic tape methods, unstandardized elastic tape application methods, and inconsistencies between studies concerning the elastic tape sessions and the duration of the elastic tape in situ. Limitations to consider on the part of the authors responsible for the synthesis of this systematic review were the inclusion of moderate impact strength articles, the methodological quality assessment tool used with the posed limitations, the search methods, and keywords used.

## Conclusions

Based on the results of this systematic review, there is no evidence supporting the effect of ETA. There is no evidence that ETA improves TRM. There is no evidence regarding application tension or direction of ETA influencing TRM. It is necessary to conduct further high-quality methodological research on the effect of ETA application on TRM to ascertain whether an effect on mobility is present. This indicates the use of more valid and reliable TRM measure instruments within a standardized measure protocol. When evaluating lumbar ETA effects on TRM and considering the psychometric quality, we cannot confirm its effects on TRM. Based on this systematic review, current lumbar ETA interventions should be questioned.

## Supplier

a. MATLAB; The Mathworks, Inc.
